# Amyloid-beta and Alzheimer’s disease: the role of neprilysin-2 in amyloid-beta clearance

**DOI:** 10.3389/fnagi.2014.00187

**Published:** 2014-08-13

**Authors:** Robert A. Marr, Daniel M. Hafez

**Affiliations:** Department of Neuroscience, Rosalind Franklin University of Medicine and ScienceNorth Chicago, IL, USA

**Keywords:** neprilysin, NEP, neprilysin-2, NEP2, amyloid hypothesis, clearance, amyloid-beta degradation, Alzheimer’s disease

## Abstract

Accumulation of the amyloid-beta (Aβ) peptide is a central factor in Alzheimer’s disease (AD) pathogenesis as supported by continuing evidence. This review concisely summarizes this evidence supporting a critical role for Aβ in AD before discussing the clearance of this peptide. Mechanisms of clearance of Aβ are critical for preventing pathological elevations in Aβ concentration. Direct degradation of Aβ by endopeptidases has emerged as one important pathway for clearance. Of particular interest are endopeptidases that are sensitive to the neprilysin (NEP) inhibitors thiorphan and phosphoramidon (i.e., are “NEP-like”) as these inhibitors induce a dramatic increase in Aβ levels in rodents. This review will focus on neprilysin-2 (NEP2), a NEP-like endopeptidase which cooperates with NEP to control Aβ levels in the brain. The evidence for the involvement of NEP2 in AD is discussed as well as the therapeutic relevance with regards to gene therapy and the development of molecular markers for the disease.

## The amyloid cascade hypothesis

### Amyloid-beta production

Alzheimer’s disease (AD) is a devastating neurodegenerative disorder that leads to behavioral, cognitive, and memory deficits. Familial AD (FAD) is inherited in an autosomal dominant pattern with symptoms typically presenting in the 4th or 5th decade of life. Sporadic late onset AD (LOAD) has a much later age of onset, usually beginning in the 7th–8th decade. Confirmed post mortem, AD pathology shows accumulations of extracellular amyloid-beta (Aβ) containing plaques and intracellular neurofibrillary *tau* tangles in the brain. The involvement of Aβ in AD is a prerequisite to the significance of Aβ clearance to AD. Therefore, we will present a rationale for the clear link between Aβ and AD progression. The formation of Aβ has been well studied (Goedert and Spillantini, 2006; Roberson and Mucke, 2006). In the amyloidogenic pathway, APP is first cleaved by β-secretase (BACE1) at amino acid *1 (of A*β*)*. When the resulting C-terminal fragment, C99, undergoes γ-secretase cleavage, it releases the amyloidogenic Aβ peptide. Key proteolytic components of γ-secretase are presenilin-1 and 2 (PS1, PS2). While various Aβ peptide lengths are produced by γ-secretase, it is Aβ_42_ and Aβ_40_ that have received the most attention. The additional two hydrophobic residues in Aβ_42_ increase its ability to aggregate, providing the scaffold for oligomeric and fibrillar forms of Aβ (Jarrett and Lansbury, 1993; Iwatsubo, 1998). It should be noted that Aβ is a naturally occurring endogenous peptide that may have normal physiological functions. For example, it has been shown that picomolar concentrations of Aβ increased LTP resulting in improved synaptic plasticity and memory (Puzzo et al., 2008, 2012; Morley et al., 2010). Therefore, pathology associated with Aβ is related to its aberrant accumulation/aggregation.

### Amyloid-beta and Alzheimer’s disease

Familial forms of early-onset AD are caused by mutations in APP, PS1, or PS2 or through increased copy number of APP (Wisniewski et al., 1985; Prasher et al., 1998; Rovelet-Lecrux et al., 2006). All of the roughly 180 mutations in PS1, 20 mutations in PS2, and 36 mutations in APP lead to elevations of total Aβ, the Aβ_42_: Aβ_40_ ratio, or its aggregation (Pimplikar, 2009). Within the Aβ sequence, point mutations, including the Arctic mutation, have been linked to increased aggregation of Aβ into protofibrils and fibrils (Clements et al., 1993, 1996; Nilsberth et al., 2001; Cheng et al., 2004; Walsh et al., 2007), as well as to reduced clearance of this peptide (Tsubuki et al., 2003; Kaden et al., 2012). However, the Arctic mutation does not affect cleavage and processing of APP; thus, this is an Aβ-only defect. Therefore, the only commonality between all the familial mutations is the effect on properties related to Aβ. To our knowledge, no other major neurodegenerative disease has familial forms that all genetically point to one common factor with the exception of monogenetic diseases like Huntington’s disease. This hypothesis is bolstered by the discovery of a protective mutation in APP in an Icelandic population that reduces BACE1 proteolysis and lowers Aβ levels (Jonsson et al., 2012). As would be consistent with the amyloid hypothesis, this mutation is associated with protection from developing AD as well as from normal cognitive decline with aging.

Unfortunately, therapies aimed at Aβ have been less than impressive in clinical trials. Most recently, Aβ-targeting monoclonal antibodies, such as bapineuzumab and solanezumab, have failed to reach their desired cognitive endpoints in trials of mild/moderate AD (Salloway et al., 2009, 2014; Farlow et al., 2012; Doody et al., 2014). Bapineuzumab did show trends for reduced Aβ_42_ levels and Aβ_42_: Aβ_40_ ratio, however, some subjects continued to demonstrate cognitive decline (Roher et al., 2013). Additionally, γ-secretase inhibitors (GSI) also failed to produce beneficial results, and were actually found to worsen cognitive function. It is believed that off-target effects, such as effects on the cleavage of notch by PS1 may have participated in producing this poor response and data suggest that the proper timing of GSIs is integral to their successful treatment of AD (Abramowski et al., 2008). In general it is unclear to what degree and for what period of time one would need to reduce Aβ levels to slow down, halt, or possibly reverse the pathology of AD. Likely, early intervention, before extensive pathological alterations occur, will be needed to effectively treat AD using anti-Aβ approaches.

### Evidence for the importance of amyloid-beta in sporadic Alzheimer’s disease

Regardless of the excuses for the failures of the many anti-Aβ clinical trials, these disappointments have understandably cast doubt over the amyloid hypothesis of AD. While the familial genetics clearly points to Aβ as a critical factor in the etiology of AD, it is conceivable that the much more common sporadic form of the disease (i.e., LOAD) has a distinct root cause(s) that is not dependent on Aβ. While this is a possibility, several lines of evidence suggest otherwise. First, these two forms of AD have very similar pathology (Lippa et al., 1996), supporting a common cause/progression. Second, if LOAD was not linked to Aβ, then polymorphisms associated with risk would be predicted to not affect Aβ. Indeed, there are many mutations affecting risk for LOAD and many of these polymorphisms are not obviously linked to Aβ; however, many others can be clearly linked to Aβ (Tanzi, 2012; Griciuc et al., 2013). What is even more important is that some of the most significant effectors of risk occur in genes strongly linked to Aβ. The most noteworthy of these are polymorphisms in the apolipoprotein E (apoE) gene with the ε4 mutation (cys112arg) multiplying risk by about 3-fold per inherited allele (Bu, 2009). ApoE can affect Aβ in multiple ways including its aggregation, clearance, and catabolism (Bales et al., 1999; Holtzman et al., 2000; Shibata et al., 2000; Fagan et al., 2002; Dolev and Michaelson, 2004; Koistinaho et al., 2004; Manelli et al., 2004; Dodart et al., 2005; Bu, 2009; Belinson et al., 2010; Hashimoto et al., 2012; Kline, 2012). Related to this, Jiang et al. (2008) showed that apoE promotes proteolytic degradation of Aβ by microglia. Another risk factor gene mutation has been discovered in ABCA7 which affects risk at a comparable level to the ε4 form of apoE (~3-fold) and has also been linked to the clearance of Aβ (Kim et al., 2013; Reitz et al., 2013). Third, if Aβ were not involved in sporadic AD then a mutation reducing Aβ production would not protect from LOAD. However, the Icelandic APP mutation (discussed above) reduces risk of the sporadic form of AD, again implicating Aβ in LOAD (Jonsson et al., 2012). These lines of evidence strongly support a critical role for Aβ in the pathogenesis of the more frequent sporadic form of the disease.

Perhaps the most common argument against the amyloid hypothesis is the fact that plaque burden correlates poorly with cognitive decline (Sorrentino et al., 2014). This discrepancy could be partially explained by current theories indicating soluble oligomeric forms of Aβ are the primary mediator of disease and not the insoluble fibrillar amyloid. However, there may be a more fundamental explanation. If Aβ is an initiator of the long and complex cascade of pathologic alterations that take place in AD, then a multitude of downstream effectors and modifying factors (including genetics, other medical conditions, and environment) would have a profound effect on the rate and severity of disease progression (Korf et al., 2004; Bennett et al., 2006; Barberger-Gateau et al., 2007; Ngandu et al., 2007; van Vliet et al., 2009; Chang et al., 2010; Rusanen et al., 2011; Reijmer et al., 2012; Tolppanen et al., 2013; Virta et al., 2013). In this scenario one would predict that Aβ would correlate more poorly (but still significantly) with cognitive decline while more downstream effectors (e.g., synaptic loss) would correlate better (Bennett et al., 2003, 2005).

## Amyloid-beta clearance

In humans, Aβ is estimated to have a physiological production rate of 7.6% per hour and a clearance rate of 8.3% per hour (Bateman et al., 2006). The various mechanisms of removal provide greater Aβ clearance than production, thus limiting its accumulation. Interestingly, human data provide evidence that accumulation in LOAD results from impaired clearance rather than increased production of Aβ (Mawuenyega et al., 2010). Using a technique of *in vivo* labeling, Mawuenyega et al. found that the clearance rate of Aβ_42_ in AD individuals was reduced to 5.3% per hour from 7.6% per hour in controls. Likewise, the Aβ_40_ clearance rate was reduced to 5.2% per hour from 7.0% per hour in controls. This finding emphasizes the importance of Aβ clearance in AD.

The proteolytic degradation of Aβ is a major route of clearance. A variety of Aβ degrading enzymes have been found and this topic has been comprehensively reviewed (Miners et al., 2011a; Nalivaeva et al., 2012). Of these enzymes, neprilysin (NEP) is considered one of the most important for the control of cerebral Aβ levels. NEP is a member of the metalloprotease 13 (M13) family of zinc metalloproteases. This 97 kD cell surface-associated enzyme functions in the periphery and central nervous system where it has been shown to degrade small peptides (Turner et al., 2001). The 50 amino acid catalytic core cleaves on the N-terminal side of hydrophobic residues (Kerr and Kenny, 1974a,b; Howell et al., 1995). Using radiolableled Aβ, Iwata et al. (2000) showed that Aβ_42_ primarily underwent degradation by NEP in their *in vivo* assay. Furthermore, application of inhibitors to NEP in rat brain produced dramatic elevations of endogenous Aβ resulting in plaque deposition. This effect was independently replicated in mice (Dolev and Michaelson, 2004; Nisemblat et al., 2008). Further supporting NEP as a critical Aβ-degrading enzyme is the observation that NEP overexpression imparts significant reductions in Aβ plaque deposition in APP-transgenic mice (Marr et al., 2003), and in some experiments, improved cognitive performance (reviewed in Marr and Spencer, 2010). It has also been shown that NEP mRNA and protein expression levels are reduced in association with age or in AD subjects (Reilly, 2001; Yasojima et al., 2001a,b; Iwata et al., 2002; Apelt et al., 2003; Caccamo et al., 2005; Maruyama et al., 2005; Wang et al., 2005, 2010); however, this notion has been seriously challenged more recently. Miners and colleagues have used a highly specific enzyme-immunocapture/activity assay to show that NEP activity levels increase with age and during the progression of AD (Miners et al., 2009, 2010, 2011b). This is similar to the consensus on most endopeptidase expression levels in association with AD (Miners et al., 2011a), and may reflect a homeostatic response to the abundance of Aβ substrate and/or to the inflammatory environment occurring in AD. Regardless, these increased endogenous levels of Aβ-degrading enzymes are ultimately insufficient to prevent the accumulation and aggregation of Aβ in AD.

Despite the data demonstrating the importance of NEP in enzymatic degradation of Aβ, other enzymes are clearly worthy of clinical study. For example, NEP knockout mice show only a moderate (1.5–2 fold) increase in Aβ levels that are far from the levels needed to induce plaque deposition, as observed with NEP inhibitors, until very advanced age (Iwata et al., 2001; Madani et al., 2006). This modest increase in Aβ raises the possibility of alternative Aβ degrading-enzymes that are likewise sensitive to NEP-inhibitors (i.e., are NEP-like).

## Neprilysin-2

**Figure 1:**
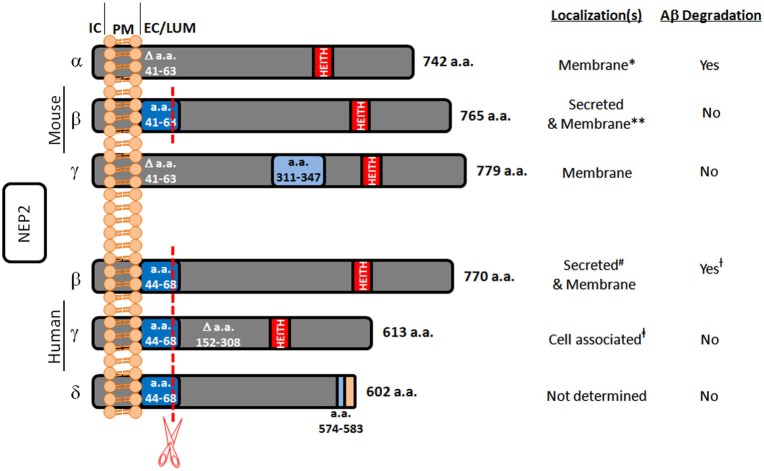
**Summary of known murine and human NEP2 splice forms**. This figure represents the protein sequences of NEP2 isoforms and summarizes their localizations and Aβ-degrading activity. The murine α-form is missing amino acids (a.a.) 41–63 which contains a furin-like processing site (red line) leaving this form membrane bound. The murine β-form contains this sequence and is secreted. The murine γ-form is missing a.a. 41–63 but contains an insertion at a.a. 311–347. All the known human forms of NEP2 contain the furin-like processing site. The β-form of human NEP2 is 77% identical to the murine β-form and is secreted. The human γ-form is missing a.a. 152–308 and is not secreted. Because of an alternate splice acceptor site, the δ-form of human NEP2 is knocked out of frame resulting in early termination and absence of the critical zinc-binding motif (HEITH). IC, intracellular; PM, plasma membrane; EC/LUM, extracellular or luminal; * associated with the membrane fraction and located inside the cell (Ikeda et al., 1999); however, *in vitro* expression did show the ability to degrade extracellular Aβ (Hafez et al., 2011); ** primarily secreted (Ghaddar et al., 2000) but also found in the membrane fraction (Ikeda et al., 1999; Raharjo et al., 2001; Shirotani et al., 2001). ^#^ associated with the membrane fraction and secreted (Whyteside and Turner, 2008); however, also shown to be expressed on the cell surface and to degrade extracellular Aβ (Huang et al., 2008); ^†^ Aβ-degrading activity found only at the extracellular surface (Huang et al., 2008); ^‡^ found in the total cell lysate (subcellular compartment localization not done) (Huang et al., 2008).

In the search for alternate Aβ degrading enzymes, NEP-like proteases are important because of their potential involvement in the spike in Aβ levels post treatment with NEP inhibitors. One such enzyme is neprilysin-2 (NEP2). NEP2 is also a zinc metalloendopeptidase belonging to the same M13 family as NEP. It has also been demonstrated in rodents that NEP2 is sensitive to the same NEP inhibitors, phosphoramidon and thiorphan (Ikeda et al., 1999; Ghaddar et al., 2000; Shirotani et al., 2001). NEP2 was first discovered while searching for an enzyme to degrade endothelin in endothelin converting enzyme-1 knockout mice and found to be a secreted peptide, termed soluble-secreted endopeptidase (SEP; Ikeda et al., 1999). Since then, it has also been referred to as neprilysin-like protein (NEPLP and Nl1) in mice and membrane-bound metalloendopeptidase-like enzyme 1 or 2 (MMEL1/2) in humans (Ghaddar et al., 2000; Bonvouloir et al., 2001; Shirotani et al., 2001). It is the closest homolog to NEP, with 55% sequence identity and similar catalytic sites. Structural modeling of NEP2 using sequence alignment and the crystal structure of NEP projects 97% identity in the active sites of these two enzymes (Voisin et al., 2004). Due to alternative splicing, murine NEP2 can exist in a membrane-bound form (mNEP2-α) or a secreted form (mNEP2-β; Figure 1). Alternative splicing also acts on the human form of NEP2 creating several isoforms. Human NEP2-β was found to be localized to both the extracellular surface and to be secreted, likely due to inefficient furan-like processing as a result of a proline residue near the processing site (P’2; Bonvouloir et al., 2001).

### NEP2 substrates and localization

In a study by Shirotani et al. (2001) using membrane-bound fractions, mNEP2-α was shown to have slower and weaker Aβ_40_ degrading properties when compared to NEP (with little to no effect on Aβ_42_), and mNEP2-β/γ had nearly undetectable activity against Aβ. However, subsequent studies using live cell assays demonstrated that mNEP2-α and hNEP2-β are able to degrade Aβ_40_, and more importantly, Aβ_42_ with comparable efficiency with NEP (Huang et al., 2008; Hafez et al., 2011; Figure 1). Interestingly, this activity was also not secreted into the cell culture medium for hNEP2-β even though the protein can be found there. hNEP2-γ did not show secreted Aβ-degrading activity or activity at the cell surface. Despite the numerous similarities between NEP2 and NEP, differences do exist. While the substrate specificities are very similar (particularly in rodents) their localizations suggest divergent roles in the central nervous system. Studies in rodents have shown that, while NEP2 is expressed at variable levels in most brain regions, it is most highly expressed in the brain stem, hypothalamus and pituitary (Ouimet et al., 2000; Facchinetti et al., 2003). Considering this localization and the substrates cleaved (e.g., gonadotropin-releasing hormone) a role in the metabolism of neuropeptides of the hypothalamo-pituitary axis has been suggested (Rose et al., 2002). Unlike NEP, NEP2 is highly expressed in the testis and brain (Bonvouloir et al., 2001), and NEP2 knockout mice do show reduced sperm function (Carpentier et al., 2004). Furthermore, hNEP2 was found to have a more restricted substrate specificity compared to hNEP with less activity against several vasoactive peptides (Whyteside and Turner, 2008). More comprehensive reviews of NEP2 are available (Marr and Spencer, 2010; Marr, 2013).

### NEP2 in Alzheimer’s disease

NEP2 knockout experiments have demonstrated the importance of NEP2 in amyloid regulation (Hafez et al., 2011). Using mice deficient for the NEP2 gene (Carpentier et al., 2004), Hafez et al. (2011) reported significant elevations in total Aβ species in the hippocampus and brainstem/diencephalon (~1.5-fold). Increases in Aβ accumulation were more dramatic in NEP2 knockout mice crossbred with APP transgenic mice. In NEP/NEP2 double-knockout mice, Aβ levels were again increased (~1.5- to 2-fold), compared with NEP^−/–^/NEP2^+/+^ controls. Treatment of these double-knockout mice with phosphoramidon-infusing pumps resulted in significant elevations in Aβ. This significant elevation in Aβ levels was also observed with intranasal treatment of phosphoramidon in both wild-type and NEP/NEP2 double-knockout mice suggesting that yet other NEP-like Aβ-degrading endopeptidases are contributing to Aβ-catabolism (Hanson et al., 2010; Hafez et al., 2011).

The importance of NEP2 was further explored in human studies. Using brain tissue from various brain regions of non-impaired, mild-cognitive impaired (MCI), and AD subjects, NEP2 mRNA expression levels in the mid-temporal gyrus were found to be lowered in women with MCI compared to non-impaired women (Huang et al., 2012). Furthermore, this altered expression was found to have a sexually dimorphic change, with males with MCI having significantly elevated NEP2 mRNA levels compared to non-impaired males in the mid-temporal and mid-frontal gyri. However, levels of NEP2 mRNA in those with AD were not different from non-impaired controls. These expression profiles closely followed what was measured for NEP in the mid-temporal gyrus. These data suggest that there is a dysregulation of NEP-like enzyme mRNA levels early in the pathogenesis of AD. Huang et al. (2012) also used an immunodepletion/activity assay to detect NEP2 activity in brain specimens and found reduced activity in association with MCI and AD regardless of sex. Therefore, this initial study suggests that NEP2 activity is reduced in association with AD, which is contrary to most of the findings with other Aβ degrading proteases (discussed above). Finally, it was also shown that NEP2 mRNA can be detected in the peripheral blood of humans. These data suggest the potential of NEP2 assays to serve as preclinical mRNA expression or enzyme activity markers for AD if expression levels in the brain mimic what is present in the blood or cerebrospinal fluid. Many neural pathologies including AD can be reflected in changes in gene expression, splicing, and protein profiles in blood and CSF, providing precedent for examining gene expression in these body fluids (Tang et al., 2005; Blennow et al., 2010; Courtney et al., 2010; Wu et al., 2011; Leuner et al., 2012; Mapstone et al., 2014). The search for new preclinical biomarkers is important given that the identification of biomarkers in AD has been relatively unsuccessful. In a recent systematic review of 59 studies, McGhee et al. (2014) found insufficient evidence to recommend using any biomarker as an outcome measure in disease progression. This highlights the importance of finding tools like NEP2 expression to detect AD early in disease progression.

## Conclusion

The impact that Alzheimer’s disease will have on the future of medicine over the next 40 years cannot be understated, and the accumulation of Aβ is currently the best theory to describe the main drive for the overall disease process. Therefore continued research into the mechanisms of Aβ clearance remains of upmost importance. The discovery of endopeptidases that degrade Aβ, such as NEP and NEP2 highlight an avenue of intervention via viral-mediated gene therapy. While NEP studies have produced encouraging results, studies utilizing NEP2 as a therapeutic agent are still warranted as NEP2 may be more selective for Aβ (Whyteside and Turner, 2008). Studies are also needed to evaluate the effectiveness of NEP2 assays as a potential molecular marker for the disease.

## Conflict of interest statement

The authors declare that the research was conducted in the absence of any commercial or financial relationships that could be construed as a potential conflict of interest.
